# Ultrasonographic Airway Measurements and Difficult Laryngoscopy in Children Undergoing Adenotonsillectomy: A Prospective Observational Study

**DOI:** 10.3390/jcm15166397

**Published:** 2026-08-19

**Authors:** Tuba Kuvvet Yoldaş, Derya Arslan Yurtlu, Zeki Tuncel Tekgül, Gaye Aydın

**Affiliations:** 1 Department of Anesthesiology and Reanimation, Izmir City Hospital, 35540 Izmir, Türkiye; darslanyurtlu@yahoo.com; 2 Department of Anesthesiology and Reanimation, Izmir Faculty of Medicine, University of Health Sciences, 35540 Izmir, Türkiye; drgayeaydin@hotmail.com; 3 Department of Anesthesiology and Reanimation, Izmir Bazekol Çiğli Hospital, 35630 Izmir, Türkiye; zekittekgul@yahoo.com; 4 Department of Anesthesiology and Reanimation, Tepecik Training and Research Hospital, 35110 Izmir, Türkiye

**Keywords:** adenotonsillectomy, ultrasonography, difficult laryngoscopy, pediatric airway, skin-to-epiglottis distance, tongue thickness

## Abstract

**Objectives:** This study aimed to evaluate the diagnostic performance of preoperative ultrasonographic airway measurements for predicting difficult laryngoscopy in pediatric patients undergoing adenotonsillectomy. **Methods:** A total of 153 pediatric patients aged 3–8 years who were scheduled for elective otorhinolaryngologic surgery were included in this prospective observational study. Patients were divided into two groups: the adenotonsillectomy group (*n* = 74) and the non-adenotonsillectomy ENT surgery group (*n* = 79). Skin-to-epiglottis distance (SED), hyomental distance (HMD) and tongue thickness (TT) were measured using ultrasonography before anesthesia induction. Laryngoscopic view was graded according to the Cormack–Lehane classification, with grades III and IV considered indicative of difficult laryngoscopy. The diagnostic performance of ultrasonographic measurements in predicting difficult laryngoscopy was evaluated using receiver operating characteristic (ROC) curve analysis. **Results:** The incidence of difficult laryngoscopy was 14.9% in the adenotonsillectomy group and 6.3% in the non-adenotonsillectomy group; however, this difference did not reach statistical significance (*p* = 0.085). In the adenotonsillectomy group, patients with difficult laryngoscopy had significantly greater skin-to-epiglottis distance and tongue thickness values (*p* = 0.002 and *p* = 0.015, respectively). No significant association was observed between hyomental distance and difficult laryngoscopy (*p* = 0.319). ROC analysis demonstrated that skin-to-epiglottis distance had the highest discriminatory performance in the adenotonsillectomy group (AUC = 0.795; 95% CI: 0.685–0.880; *p* = 0.004). **Conclusions:** Skin-to-epiglottis distance and tongue thickness were associated with difficult laryngoscopy in pediatric patients undergoing adenotonsillectomy. Among the evaluated ultrasonographic parameters, skin-to-epiglottis distance demonstrated the highest diagnostic performance. Preoperative ultrasonographic airway assessment may provide valuable information for identifying children at increased risk of difficult laryngoscopy. These findings should be confirmed in larger multicenter studies.

## 1. Introduction

Unexpected difficult laryngoscopy (DL) is one of the most important anesthesia-related complications in pediatric patients and may result in severe hypoxia, bradycardia, and even mortality [1]. The reported incidence of difficult laryngoscopy in children ranges from approximately 1% to 8%, depending on patient age, underlying pathology, surgical population, and the definition of difficult laryngoscopy used, with substantially higher rates observed in selected high-risk populations [2,3]. Consequently, accurate preoperative airway assessment is essential for identifying children at increased risk of difficult laryngoscopy and optimizing airway management strategies [2,4].

Conventional airway assessment methods, including the Mallampati classification, thyromental distance, and mouth opening, have shown variable predictive performance in pediatric patients [5]. Although limited cooperation may reduce the reliability of these assessments, the distinctive anatomical characteristics of the pediatric airway—including a relatively larger tongue, a more cephalad and anteriorly positioned larynx, and ongoing craniofacial development—may also influence their diagnostic performance because most bedside airway assessment tests were originally developed and validated in adults [6].

In recent years, point-of-care ultrasonography (POCUS) has emerged as a promising adjunct to conventional airway assessment by providing rapid, non-invasive visualization of upper airway structures [1,7]. Ultrasonographic parameters such as tongue thickness (TT), hyomental distance (HMD), and skin-to-epiglottis distance (SED) have shown potential for predicting difficult laryngoscopy [8,9]. However, the routine use of airway ultrasonography may be limited by equipment availability, operator expertise, and the additional time required for image acquisition, particularly in high-turnover pediatric ENT operating rooms. Therefore, identifying informative ultrasonographic parameters may help improve the clinical utility of airway ultrasound.

Several studies in adults have demonstrated that ultrasonographic measurements may help predict difficult laryngoscopy; however, evidence in pediatric patients remains limited, and their diagnostic value has not yet been clearly established [10,11].

Children with adenotonsillar hypertrophy constitute a clinically important subgroup because enlargement of the tonsillar and adenoid tissues alters upper airway anatomy by causing airway narrowing and posterior displacement of the tongue base. These anatomical changes may influence the performance of conventional bedside airway assessment methods and may also increase the risk of difficult laryngoscopy [1]. Nevertheless, studies evaluating ultrasonographic airway measurements in this population remain scarce. We therefore hypothesized that the diagnostic performance of ultrasonographic airway measurements may differ between children undergoing adenotonsillectomy and those undergoing other elective ENT procedures.

Therefore, the aim of the present study was to evaluate the diagnostic performance of preoperative ultrasonographic airway measurements for predicting difficult laryngoscopy in children with adenotonsillar hypertrophy and to determine whether their predictive performance differs from that observed in children undergoing elective ENT surgery without upper airway obstruction.

## 2. Materials and Methods

### 2.1. Study Design and Ethical Approval

This study was designed as a prospective, observational investigation conducted at a single tertiary care center. Ethical approval was granted by the Institutional Ethics Committee (Approval No. 2025/42), and the study protocol complied with the ethical standards outlined in the Declaration of Helsinki. The study was prospectively registered at ClinicalTrials.gov (NCT07057661). Written informed consent was obtained from the parents or legal guardians of all children before participation. Recruitment was carried out between June 2025 and March 2026, and all eligible patients presenting during this period were consecutively included in the study.

### 2.2. Patient Population

Children aged 3–8 years classified as ASA physical status I–II and undergoing elective surgery were considered eligible for inclusion. After enrollment, patients were categorized into two groups according to the presence or absence of adenotonsillar hypertrophy. This grouping was performed to determine whether adenotonsillar hypertrophy influences the diagnostic performance of ultrasonographic airway measurements for predicting difficult laryngoscopy.

1. Adenotonsillectomy Group (*n* = 74): Patients scheduled for adenotonsillectomy or tonsillectomy due to adenotonsillar hypertrophy.
2. Non-adenotonsillectomy ENT Surgery Group (*n* = 79): Patients undergoing elective otorhinolaryngologic procedures without upper airway obstruction, including tympanoplasty, tympanostomy tube insertion, otoplasty, and other minor otologic surgeries.

Exclusion criteria included a history of congenital head, neck, or facial anomalies; ASA physical status III–V; emergency surgery; planned use of alternative airway management techniques other than orotracheal intubation (e.g., supraglottic airway devices); and the use of anesthetic techniques different from the study protocol.

### 2.3. Preoperative Assessment and Ultrasonographic Measurements

Baseline characteristics, including age, sex, and body mass index (BMI), were documented before surgery. As part of the conventional airway assessment, the modified Mallampati score was determined according to the patient’s level of cooperation, taking into consideration the limitations of this assessment in younger children.

**Figure 1:**
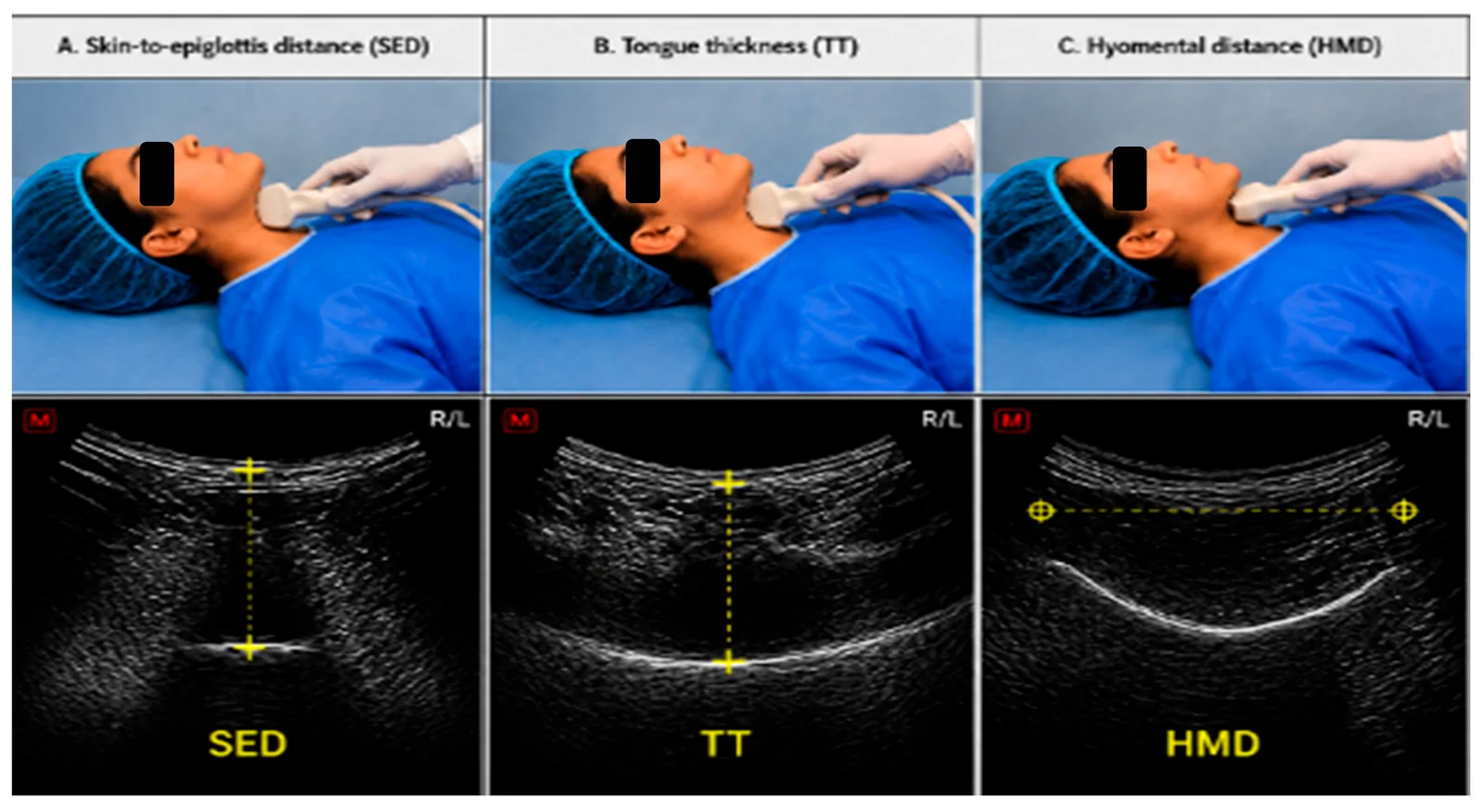
Positioning of the ultrasound probe and corresponding sonographic images used for measurement of (**A**) skin-to-epiglottis distance (SED), (**B**) tongue thickness (TT), and (**C**) hyomental distance (HMD). The yellow calipers and measurement lines indicate the anatomical distances measured for each ultrasonographic parameter. Clinical photographs and ultrasonographic images were obtained by the authors. Probe positioning and measurement techniques were prepared according to the methodology described by Zheng et al. [1].

Before anesthesia induction, all patients underwent ultrasonographic airway assessment with the head in maximal extension. Airway ultrasonography was performed by a single anesthesiologist experienced in ultrasound-guided airway assessment using a 6–13 MHz high-frequency linear probe (LOGIQ, GE HealthCare, Chicago, IL, USA). A predefined scanning protocol based on the methodology described by Zheng et al. [1] was followed to ensure consistency throughout the study. Representative probe positioning and corresponding ultrasonographic images are shown in Figure 1.

For HMD assessment, the linear probe was placed beneath the chin in the midsagittal plane. It was then rotated 90° and positioned transversely in the submental region for TT assessment. Finally, the probe was positioned transversely at the level of the thyrohyoid membrane for SED assessment.

SED was defined as the shortest distance between the skin and the anterior surface of the epiglottis at the level of the thyrohyoid membrane. HMD was defined as the distance between the mentum and the hyoid bone with the head in maximal extension. TT was defined as the maximum vertical thickness of the tongue on the ultrasonographic image obtained from the submental region.

### 2.4. Anesthesia Management and Intraoperative Assessment

To ensure uniform perioperative management, all participants received the same general anesthesia regimen throughout the study. Following routine monitoring, anesthesia was induced with propofol (2–3 mg/kg) and fentanyl (1 μg/kg). Neuromuscular blockade was achieved with rocuronium (0.6 mg/kg), and orotracheal intubation was subsequently performed after confirmation of adequate neuromuscular blockade. Adequate neuromuscular blockade was confirmed clinically before laryngoscopy by the absence of spontaneous respiratory efforts, complete jaw relaxation, and satisfactory chest compliance during controlled mask ventilation after allowing sufficient onset time for rocuronium. Anesthesia was continued with sevoflurane (2–3%) administered in a 1:1 oxygen–air mixture. Mask ventilation was graded according to the Han grading system, and difficult mask ventilation (DMV) was defined as Han grade III or IV [12].

Following confirmation of adequate neuromuscular blockade, direct laryngoscopy was undertaken and the laryngeal view was categorized according to the Cormack–Lehane grading system. All airway assessments were conducted by a senior anesthesiologist experienced in pediatric airway management. To reduce observer bias, the clinician responsible for laryngoscopic grading had no access to the ultrasonographic airway measurements obtained before induction of anesthesia. Laryngoscopic views corresponding to Cormack–Lehane grades III and IV were classified as difficult laryngoscopy. All patients initially underwent direct laryngoscopy using a Macintosh blade. In patients requiring more than one intubation attempt, subsequent intubation was performed using a video laryngoscope with a standard Macintosh-type blade. The size of the Macintosh blade and the endotracheal tube was selected according to the patient’s age and body size.

### 2.5. Outcome Measurements

#### 2.5.1. Primary Outcome

The primary outcome was the diagnostic performance of preoperative ultrasonographic airway measurements (SED, TT, and HMD) for predicting difficult laryngoscopy in children undergoing adenotonsillectomy for adenotonsillar hypertrophy.

#### 2.5.2. Secondary Outcomes

The secondary outcomes were the incidence of difficult laryngoscopy and differences in ultrasonographic airway measurements (SED, TT, and HMD) between the adenotonsillectomy and non-adenotonsillectomy groups.

### 2.6. Statistical Analysis

Sample size calculation for the comparison of two groups was performed using G*Power version 3.1.9.4. Since no previous study had specifically evaluated the relationship between ultrasonographic airway measurements and difficult laryngoscopy in children with adenotonsillar hypertrophy, a medium effect size (effect size = 0.50) was assumed, taking into consideration the findings of the pediatric airway ultrasonography study by Zheng et al. With a significance level of α = 0.05 and a statistical power (1 − β) of 80%, the minimum required sample size was calculated as 51 patients per group, yielding a total sample size of 102 patients.

All statistical procedures were conducted with IBM SPSS Statistics for Windows, Version 20.0 (IBM Corp., Armonk, NY, USA). Data distribution was evaluated using the Kolmogorov–Smirnov and Shapiro–Wilk tests. Continuous variables demonstrating normal distribution are presented as mean ± standard deviation, while skewed variables are reported as median (interquartile range).

Comparisons of continuous variables were performed according to data distribution. Variables showing a normal distribution were analyzed using Student’s *t*-test, whereas the Mann–Whitney U test was used for variables that did not meet normality assumptions. Categorical data were summarized as frequencies and percentages and analyzed using either the chi-square test or Fisher’s exact test, as appropriate.

The diagnostic accuracy of ultrasonographic airway measurements for identifying difficult laryngoscopy was assessed using receiver operating characteristic (ROC) curve analysis. For each parameter, the area under the curve (AUC), corresponding 95% confidence intervals, optimal threshold values, sensitivity, and specificity were determined. Statistical significance was defined as a two-sided *p*-value of less than 0.05.

## 3. Results

**Figure 2:**
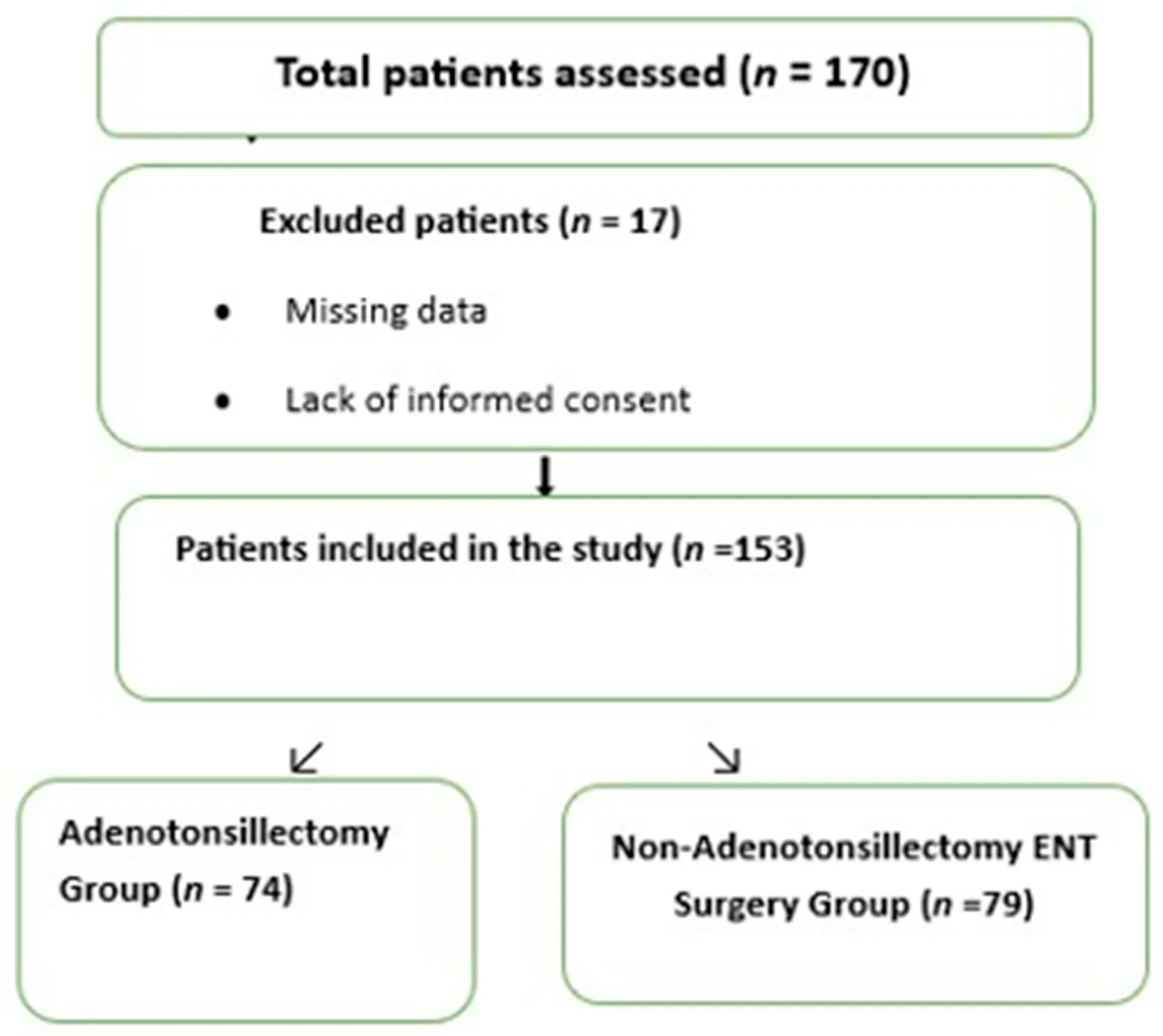
Patient Flow Diagram.

A total of 153 pediatric patients were included in the study. Of these, 74 patients were included in the adenotonsillectomy group and 79 in the non-adenotonsillectomy ENT surgery group. Figure 2 summarizes the recruitment pathway of the study population and the allocation of participants to each study group. Baseline demographic and anthropometric characteristics, including age, weight, height, and BMI, were similar across the study groups (all *p* > 0.05). However, the distribution of ASA physical status differed significantly between the groups (*p* = 0.029). Modified Mallampati scores were comparable between the groups (*p* = 0.968). In contrast, the distribution of Cormack–Lehane grades differed significantly between the groups (*p* = 0.005), with higher Cormack–Lehane grades occurring more frequently in the adenotonsillectomy group. Although the incidence of difficult laryngoscopy was higher in the adenotonsillectomy group than in the non-adenotonsillectomy group (14.9% vs. 6.3%), the difference did not reach statistical significance (*p* = 0.085). Difficult mask ventilation (Han grade III–IV) was observed in 10 (13.5%) patients in the adenotonsillectomy group and 4 (5.1%) patients in the non-adenotonsillectomy group, with no statistically significant difference between the groups (*p* = 0.070) (Table 1).

With respect to ultrasonographic measurements, tongue thickness was significantly greater in the adenotonsillectomy group compared with the non-adenotonsillectomy group (3.99 ± 0.31 cm vs. 3.52 ± 0.30 cm, *p* < 0.001). Similarly, the skin-to-epiglottis distance was significantly higher in the adenotonsillectomy group (*p* = 0.049). No significant difference was observed in hyomental distance between the groups (*p* = 0.730) (Table 1).

Within the adenotonsillectomy group, patients with difficult laryngoscopy were significantly older than those without difficult laryngoscopy (*p* = 0.045). In addition, both skin-to-epiglottis distance (*p* = 0.002) and tongue thickness (*p* = 0.015) were significantly greater in patients with difficult laryngoscopy. No significant differences were observed regarding hyomental distance, BMI, weight, height, or modified Mallampati score (*p* > 0.05 for all comparisons). The number of intubation attempts was significantly higher in patients with difficult laryngoscopy (*p* < 0.001) (Table 2).

**Figure 3:**
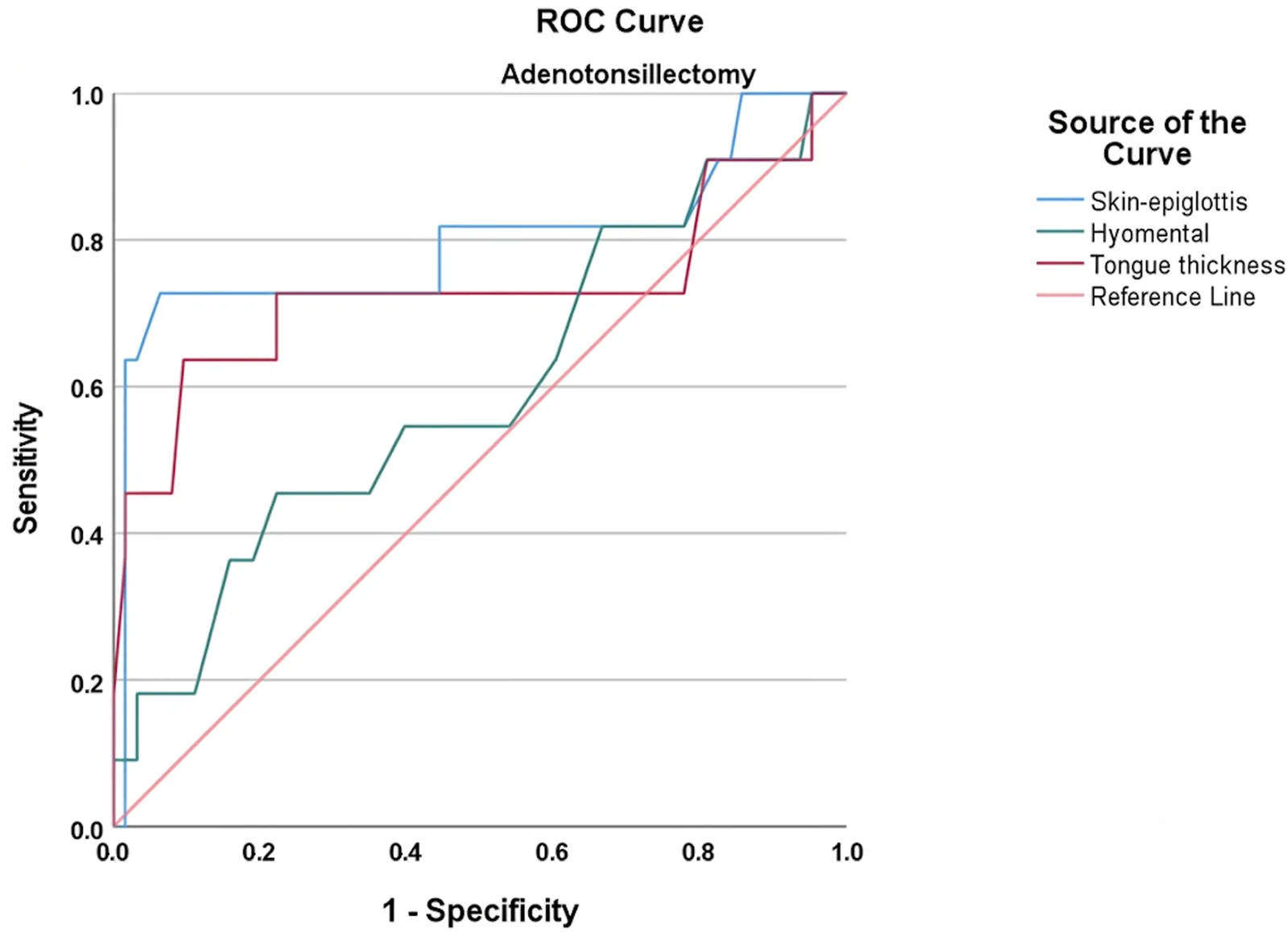
ROC curves of ultrasonographic airway measurements for predicting difficult laryngoscopy in the adenotonsillectomy group. Skin-to-epiglottis distance demonstrated the highest diagnostic performance (AUC = 0.795), followed by tongue thickness (AUC = 0.730). Hyomental distance showed no significant predictive value (AUC = 0.595).

ROC curve analysis demonstrated that, in the overall study population, both skin-to-epiglottis distance (AUC = 0.731, *p* = 0.007) and tongue thickness (AUC = 0.706, *p* = 0.012) showed significant discriminatory ability for difficult laryngoscopy. In the adenotonsillectomy group, skin-to-epiglottis distance demonstrated the highest diagnostic performance (AUC = 0.795, *p* = 0.004). The optimal cut-off value for this parameter was >1.54 cm, yielding a sensitivity of 72.7% and a specificity of 93.7%. Tongue thickness also showed significant discriminatory ability (AUC = 0.730, *p* = 0.046). In contrast, hyomental distance was not significantly associated with difficult laryngoscopy (*p* > 0.05) (Table 3, Figure 3). In the non-adenotonsillectomy group, none of the ultrasonographic parameters demonstrated a significant association with difficult laryngoscopy.

## 4. Discussion

In this prospective study, important findings were obtained suggesting that SED and TT may be associated with difficult laryngoscopy in pediatric patients undergoing adenotonsillectomy. Among all evaluated ultrasonographic parameters, SED demonstrated the highest discriminatory performance on ROC analysis. In contrast, HMD, which is frequently used in adult studies, was not significantly associated with difficult laryngoscopy in our pediatric cohort.

Preoperative prediction of a difficult airway remains a major clinical challenge in pediatric anesthesia. Although airway ultrasonography has increasingly been used for this purpose in recent years, the available evidence in children remains limited and inconsistent compared with that in adults [3,6]. For example, Zheng et al. reported that SED may be useful for predicting difficult laryngoscopy in children [1], whereas Kaya et al. failed to demonstrate a significant association between ultrasonographic measurements and difficult laryngoscopy in their prospective study [13].

The findings obtained in the non-adenotonsillectomy ENT surgery group in our study were similar to those reported by Kaya et al. In contrast, the observation that SED and TT were significantly associated with difficult laryngoscopy only in the adenotonsillectomy group represents one of the most distinctive findings of our study. This suggests that the performance of ultrasonographic airway parameters may depend not only on age but also on specific pathological conditions, such as adenotonsillar hypertrophy (ATH), that directly alter upper airway anatomy.

ATH is the most common cause of upper airway obstruction in children and is characterized by snoring, mouth breathing, and increased upper airway resistance. Chronic tissue hypertrophy not only causes mechanical obstruction but also leads to long-term alterations in craniofacial development, breathing patterns, and upper airway morphology. Jaiswal et al. demonstrated that mouth breathing and secondary craniofacial changes occur more frequently in children with ATH. In addition, maxillomandibular retrusion, increased vertical facial growth patterns, and narrowing of the pharyngeal airway have been described in this population [14]. Feng et al. further reported that severe adenoid and tonsillar hypertrophy is directly associated with the severity of obstructive sleep apnea (OSA) [15].

These findings suggest that these chronic anatomical and functional alterations of the upper airway may enhance the discriminatory ability of ultrasonographic measurements by affecting tongue base position and the projection of soft tissues surrounding the epiglottis. Indeed, while the overall incidence of difficult laryngoscopy in our study was 10.5%, this rate increased to 14.9% in the adenotonsillectomy group composed of children with ATH. The lower overall incidence of difficult laryngoscopy reported by Zheng et al. (4.8%) [1] may be explained by differences in the prevalence and distribution of ATH between the study populations. Furthermore, patients with difficult laryngoscopy in our study were significantly older. This finding may be related to the progressive nature of adenotonsillar hypertrophy and its increasing impact on upper airway anatomy with age. However, since our study was not specifically designed to investigate this relationship, this finding should be interpreted with caution.

Several adult studies have shown that tongue thickness, hyomental distance in extension, and anterior neck soft tissue thickness are useful predictors of difficult airway management [16,17,18,19,20]. The relatively stable anatomy of the adult airway and the direct effect of increased soft tissue mass on glottic visualization may explain the predictive value of these parameters. In contrast, the pediatric airway undergoes continuous growth and development, with substantial age-related variations in tissue compliance and anatomical proportions. Consequently, cut-off values established in adults cannot be directly extrapolated to children.

Among the ultrasonographic measurements examined in children with adenotonsillar hypertrophy, SED demonstrated the greatest discriminatory performance for difficult laryngoscopy, consistent with the findings of Zheng et al. [1]. More recently, Altınsoy et al. also reported that SED was the most accurate ultrasonographic predictor of difficult intubation in obese adults, further supporting the concept that SED reflects clinically relevant anterior neck soft-tissue anatomy despite differences in patient populations [21]. Similarly, TT was significantly greater only in adenotonsillectomy patients with difficult laryngoscopy. While Yao et al. identified TT as an independent predictor of difficult laryngoscopy in adults [18], Zheng et al. failed to demonstrate a significant association in pediatric patients [1]. The association observed in our study may be related to chronic mouth breathing associated with ATH, resulting in inferior and anterior postural displacement of the tongue, tongue base hypertrophy, or adaptive changes in surrounding soft tissues.

Although lingual tonsil hypertrophy was not specifically evaluated in the present study, enlargement of the palatine and lingual tonsils has been reported to modify upper airway geometry and adjacent soft tissue anatomy. These anatomical alterations may influence ultrasonographic measurements such as TT and SED by reducing the retrolingual space, displacing the tongue base posteriorly, and increasing the depth of the pre-epiglottic soft tissues. This interpretation is consistent with previous reports describing the effects of tonsillar hypertrophy on upper airway morphology [22,23,24].

In addition to these anatomical considerations, skin-to-epiglottis distance (SED) has been suggested as a useful ultrasonographic parameter for predicting difficult laryngoscopy in both adult and pediatric populations [1,17,21]. In children with adenotonsillar hypertrophy, enlargement of the adenoids and palatine tonsils may alter upper airway anatomy and increase the depth of the soft tissues anterior to the epiglottis, which may contribute to higher SED values. Consequently, increased SED may reflect anatomical changes that are associated with more limited glottic visualization during direct laryngoscopy. This may partly explain why SED demonstrated good discriminatory performance in our adenotonsillectomy cohort. Our findings therefore suggest that SED may be particularly useful in children with adenotonsillar hypertrophy. In contrast, Kaya et al. did not observe a significant association between ultrasonographic measurements and difficult laryngoscopy [13], which may be related to differences in study populations and the prevalence of adenotonsillar hypertrophy. In addition, previous adult studies have shown that increased SED is associated not only with difficult laryngoscopy but also with difficult mask ventilation, suggesting that this measurement may reflect clinically relevant anterior neck soft tissue characteristics [25,26]. However, although difficult mask ventilation was recorded in our study, no significant difference was observed between patients with and without difficult laryngoscopy.

The literature regarding HMD remains heterogeneous. Although Zheng et al. suggested that HMD may be useful for predicting difficult laryngoscopy in children [1], neither our study nor the study by Kaya et al. [13] demonstrated a significant association between HMD and difficult laryngoscopy. Taken together, these findings suggest that the predictive performance of HMD may vary across different pediatric populations and clinical settings [1,13].

Several factors may have contributed to the lack of a significant association between HMD and difficult laryngoscopy in our cohort. First, the pediatric airway differs substantially from the adult airway, with a more cephalad and anteriorly positioned larynx, a relatively larger tongue, and continuous craniofacial development throughout childhood [6]. These anatomical characteristics may limit the ability of the linear distance between the mentum and hyoid bone to accurately reflect the complexity of the pediatric airway during direct laryngoscopy. Accordingly, ultrasonographic parameters such as skin-to-epiglottis distance (SED) and tongue thickness (TT), which more directly assess anterior soft-tissue anatomy, may provide additional information regarding glottic visualization than HMD [10,16]. Furthermore, reliable HMD measurement depends on achieving standardized maximal head extension. In young children, maintaining a consistent head position before the induction of anesthesia may be technically challenging because of limited cooperation, greater tissue compliance, and age-related anatomical variability. Even minor variations in head position could potentially influence HMD measurements and contribute to measurement variability. In addition, recent reviews of airway ultrasonography have emphasized that upper airway ultrasound measurements remain operator-dependent and may be influenced by patient positioning, probe orientation, and measurement standardization, all of which could affect their diagnostic performance [9,27].

### Strengths and Limitations

The major strength of our study is that it specifically evaluated children undergoing adenotonsillectomy as a distinct subgroup, making it one of the few prospective studies to investigate the influence of ATH on the predictive value of airway ultrasonographic measurements.

However, several limitations should be acknowledged. First, the single-center design and relatively limited sample size may have reduced the statistical power of certain subgroup analyses, particularly given the small number of patients with difficult laryngoscopy. Second, because only patients undergoing elective ENT surgery were included, the findings cannot be directly generalized to the broader pediatric population. Third, OSA status and severity were not prospectively assessed using standardized methods such as polysomnography. Because OSA is closely associated with adenotonsillar hypertrophy and upper airway collapsibility, future studies incorporating objective OSA assessment may further clarify the relationship between OSA, ultrasonographic airway measurements, and difficult laryngoscopy. Finally, the operator-dependent nature of airway ultrasonography and the challenges associated with maintaining optimal positioning in young children may have introduced minor measurement variability.

## 5. Conclusions

In conclusion, the diagnostic performance of ultrasonographic airway measurements appeared to differ according to the presence of adenotonsillar hypertrophy. SED and TT were associated with difficult laryngoscopy only in children undergoing adenotonsillectomy, whereas no significant association was observed in children undergoing other elective ENT procedures without upper airway obstruction. Of the evaluated ultrasonographic parameters, SED demonstrated the highest diagnostic performance and may provide useful additional information during preoperative airway assessment in this population. These findings suggest that airway ultrasonography may have greater clinical utility in children with adenotonsillar hypertrophy than in those without upper airway obstruction.

## Figures and Tables

**Table 1:** Demographic characteristics, airway assessment findings, and ultrasonographic measurements of the study groups.

Variable	Adenotonsillectomy Group (*n* = 74)	Other ENT Surgery Group (*n* = 79)	*p*-Value
Sex (Male/Female), *n*	51/23	59/20	0.428
ASA physical status (I/II), *n*	64/10	57/22	0.029 *
Modified Mallampati score (I/II), *n*	56/18	60/19	0.968
Difficult laryngoscopy, *n* (%)	11 (14.9)	5 (6.3)	0.085
Difficult mask ventilation, *n* (%)	10 (13.5)	4 (5.1)	0.070
Cormack–Lehane grade (I/II/III/IV), *n*	25/38/10/1	49/25/5/0	0.005 *
Age (years)	6.2 ± 1.61	6.39 ± 1.39	0.524
Weight (kg)	22.22 ± 7.03	21.84 ± 4.91	0.483
Height (m)	1.16 ± 0.11	1.17 ± 0.08	0.383
Body mass index (kg/m^2^)	16.5 ± 3.83	15.7 ± 2.12	0.463
Skin-to-epiglottis distance (cm)	1.36 ± 0.19	1.34 ± 0.09	0.049 *
Hyomental distance (cm)	4.04 ± 0.18	4.06 ± 0.19	0.730
Tongue thickness (cm)	3.99 ± 0.31	3.52 ± 0.30	<0.001 *

Data are presented as mean ± standard deviation (SD), median (interquartile range [IQR]), or number (*n*), as appropriate. ASA: American Society of Anesthesiologists; BMI: body mass index; CL: Cormack–Lehane. * Indicates a statistically significant difference (*p* < 0.05).

**Table 2:** Comparison of Clinical and Ultrasonographic Variables According to the Presence of Difficult Laryngoscopy in the Adenotonsillectomy Group.

Variable	No Difficult Laryngoscopy (*n* = 63)	Difficult Laryngoscopy (*n* = 11)	*p*-Value
Sex (Male/Female), *n*	43/20	8/3	1.000
ASA (I/II), *n*	53/10	11/0	0.341
Modified Mallampati Score (I/II), *n*	47/16	9/2	1.000
Difficult Mask Ventilation (No/Yes), *n*	56/7	8/3	0.163
Cormack–Lehane Grade (I/II/III/IV), *n*	25/37/1/0	0/1/9/1	<0.001 *
Age (years), mean ± SD	6.05 ± 1.56	7.09 ± 1.70	0.045 *
Weight (kg), mean ± SD	21.71 ± 6.87	25.09 ± 7.56	0.123
Height (m), mean ± SD	1.15 ± 0.11	1.20 ± 0.11	0.126
Body Mass Index (kg/m^2^), mean ± SD	16.37 ± 3.77	17.25 ± 4.32	0.361
Skin-to-Epiglottis Distance (cm), mean ± SD	1.34 ± 0.18	1.49 ± 0.19	0.002 *
Hyomental Distance (cm), mean ± SD	4.03 ± 0.18	4.09 ± 0.20	0.319
Tongue Thickness (cm), mean ± SD	3.95 ± 0.27	4.21 ± 0.41	0.015 *
Number of Intubation Attempts, mean ± SD	1.08 ± 0.27	1.64 ± 0.67	<0.001 *

Data are presented as mean ± standard deviation (SD) or number (*n*), as appropriate. Difficult laryngoscopy was defined as Cormack–Lehane grade III–IV view. BMI: body mass index; ASA: American Society of Anesthesiologists. * Indicates a statistically significant difference (*p* < 0.05).

**Table 3:** Diagnostic Performance of Ultrasonographic Measurements for Predicting Difficult Laryngoscopy.

Parameter	Cut-Off Value (cm)	Sensitivity (%)	Specificity (%)	AUC (95% CI)	*p*-Value
Overall Population					
Skin-to-Epiglottis Distance	>1.54	50.0	97.1	0.731 (0.653–0.799)	0.007 *
Tongue Thickness	>4.25	37.5	95.6	0.706 (0.627–0.776)	0.012 *
Hyomental Distance	>4.13	50.0	71.5	0.584 (0.520–0.663)	0.298
Adenotonsillectomy Group					
Skin-to-Epiglottis Distance	>1.54	72.7	93.7	0.795 (0.685–0.880)	0.004 *
Tongue Thickness	>4.25	63.6	90.5	0.730 (0.614–0.827)	0.046 *
Hyomental Distance	>4.13	45.5	77.8	0.595 (0.474–0.707)	0.361

AUC: area under the curve; CI: confidence interval; PPV: positive predictive value; NPV: negative predictive value. Optimal cut-off values were determined using ROC curve analysis. * Indicates a statistically significant difference (*p* < 0.05).

## Data Availability

The data presented in this study are available from the corresponding author upon reasonable request.
